# Gain and Loss of Multiple Genes During the Evolution of *Helicobacter pylori*

**DOI:** 10.1371/journal.pgen.0010043

**Published:** 2005-10-07

**Authors:** Helga Gressmann, Bodo Linz, Rohit Ghai, Klaus-Peter Pleissner, Ralph Schlapbach, Yoshio Yamaoka, Christian Kraft, Sebastian Suerbaum, Thomas F Meyer, Mark Achtman

**Affiliations:** 1 Department of Molecular Biology, Max-Planck-Institut für Infektionsbiologie, Berlin, Germany; 2 Institut für Medizinische Mikrobiologie, Justus-Liebig-Universität, Giessen, Germany; 3 Core Facility Bioinformatics, Max-Planck-Institut für Infektionsbiologie, Berlin, Germany; 4 Functional Genomics Center Zurich, ETH Zurich/University of Zurich, Zurich, Switzerland; 5 Department of Medicine, M.E. DeBakey Veterans Affairs Medical Center and Baylor College of Medicine, Houston, Texas, United States of America; 6 Medizinische Hochschule Hannover, Institut für Medizinische Mikrobiologie und Krankenhaushygiene, Hannover, Germany; National Institute of Genetics, Japan

## Abstract

Sequence diversity and gene content distinguish most isolates of *Helicobacter pylori*. Even greater sequence differences differentiate distinct populations of *H. pylori* from different continents, but it was not clear whether these populations also differ in gene content. To address this question, we tested 56 globally representative strains of *H. pylori* and four strains of *Helicobacter acinonychis* with whole genome microarrays. Of the weighted average of 1,531 genes present in the two sequenced genomes, 25% are absent in at least one strain of *H. pylori* and 21% were absent or variable in *H. acinonychis.* We extrapolate that the core genome present in all isolates of *H. pylori* contains 1,111 genes. Variable genes tend to be small and possess unusual GC content; many of them have probably been imported by horizontal gene transfer. Phylogenetic trees based on the microarray data differ from those based on sequences of seven genes from the core genome. These discrepancies are due to homoplasies resulting from independent gene loss by deletion or recombination in multiple strains, which distort phylogenetic patterns. The patterns of these discrepancies versus population structure allow a reconstruction of the timing of the acquisition of variable genes within this species. Variable genes that are located within the *cag* pathogenicity island were apparently first acquired en bloc after speciation. In contrast, most other variable genes are of unknown function or encode restriction/modification enzymes, transposases, or outer membrane proteins. These seem to have been acquired prior to speciation of *H. pylori* and were subsequently lost by convergent evolution within individual strains. Thus, the use of microarrays can reveal patterns of gene gain or loss when examined within a phylogenetic context that is based on sequences of core genes.

## Synopsis

The Gram-negative pathogenic bacterium *Helicobacter pylori* colonizes the stomach of 50% of mankind and has probably infected humans since their origins. Due to geographic isolation and frequent local recombination, phylogeographic differences within *H. pylori* have arisen, resulting in multiple populations and subpopulations that mirror ancient human migrations and genetic diversity. We have examined the gene content of representatives of these populations by whole genome microarrays. Only 1,111 genes are predicted to exist in all *H. pylori* of the 1,531 that are present on average in two sequenced genomes. Missing genes fall into two classes: one class contains genes within the *cag* pathogenicity island that was acquired en bloc after speciation and is present only in particular populations. The second class contains a variety of genes whose function may be unimportant for the cell and that were acquired prior to speciation. Their absence in individual isolates reflects convergent evolution through gene loss. Thus, patterns of gene gain or loss can be identified by whole genome microarrays within a phylogenetic context that can be supplied by sequences of genes from the core genome.

## Introduction

The Gram-negative pathogenic bacterium *Helicobacter pylori* colonizes the stomach of 50% of mankind [1] and has probably infected humans since their origins [2]. *H. pylori* is acquired during childhood by intrafamilial transmission [3,4], and infection continues lifelong in the absence of antibiotic therapy. It has been postulated [5] that this lifelong association is accompanied by the selection of well-adapted, host-specific variants with particular patterns of expression of adhesins [6,7] and other surface molecules [8,9]. Recombination is frequent during transient colonization with multiple strains due to DNA transformation, resulting in variants within individual hosts that differ in sequence content [10] and genomic composition [11]. As a result of frequent recombination, almost all *H. pylori* isolates from unrelated hosts possess unique sequences [12], unlike other bacteria, where identical sequences of core housekeeping genes are found in multiple isolates [13]. *H. pylori* differ between individual hosts [12,14–18], but even greater differences are found when sequences are compared with isolates from different continents [18–20], possibly reflecting genetic drift during geographic isolation [12], as well as adaptation to genetic differences between different ethnic groups of humans [7,19].

*H. pylori* have been grouped into multiple populations and subpopulations on the basis of sequence differences in seven core genes [12]. These were designated hpEurope, which is common in Europe and countries colonized by Europeans; hpAfrica1, with subpopulations hspWAfrica (West Africa, South Africa, and the Americas) and hspSAfrica (South Africa); hpEastAsia, with subpopulations hspMaori (Polynesians), hspAmerind (Native Americans), and hspEAsia (East Asia); and hpAfrica2, which is very distinct and has been isolated only in South Africa [12]. Recent work (B.Linz, unpublished data) has defined an additional *H. pylori* population, hpAsia2, which is found in Central and South Asia.

Two genome sequences are available, namely from strains 26695 [21] and J99 [22]. These strains belong to the hpEurope population and the hspWAfrica subpopulation of hpAfrica1, respectively [12]. Of their coding sequences (CDSs), 6% are genome-specific, due to multiple insertions and deletions. Half of these genome-specific CDSs are located in two regions called plasticity zones 1 and 2 [22] that are located 600 kilobases (kb) apart in strain 26695 but are joined in strain J99. These plasticity zone(s) cover a total of 68 kb in 26695 and 45 kb in J99. In addition to plasticity zones 1 and 2, some strains of *H. pylori* possess a 37 kb pathogenicity island, called the *cag* pathogenicity island (*cag* PAI), while others do not [23]. In microarray studies, 22% of the CDSs in the two genomic sequences were absent in at least one of 15 *H. pylori* strains [24]. It seemed possible that the differential presence or absence of multiple CDSs might correlate with *H. pylori* population structure, but this question has not yet been addressed.

Bacterial population structures differ with the taxa under investigation [25,26]. Some species, such as *Mycobacterium tuberculosis* and *Yersinia pestis,* are of such recent origin that very little sequence diversity has yet accumulated [27,28]. In such largely clonal species, only a few CDSs are variably present among different isolates [29–32], and the absence of individual CDSs correlates with sequence differences between strains of *Y. pestis* [28]. Similarly, microarray analysis of *Listeria monocytogenes* identified groups of CDSs whose presence correlated with subdivisions according to serotyping [33]. Microarray analyses of common serovars of *Salmonella enterica* grouped isolates together that were known to be closely related according to multilocus enzyme electrophoresis [34], with exceptions that may reflect horizontal genetic exchange and subsequent selection. Based on these results, it might be anticipated that whole genome comparisons based on microarrays would not only provide inferences about phenotypic differences within a species but also reveal the general population structure of the bacteria under investigation. However, population structure according to microarrays has not yet been investigated in any species in which a global population structure has already been determined by established methods. And the accuracy of population structures according to microarrays has not been examined for bacteria with high sequence diversity and frequent homologous recombination, such as *Neisseria meningitidis* [35] or *H. pylori.* We addressed these issues by performing microarray analyses with isolates that are representative of the global diversity of *H. pylori*. In order to provide a close out-group for resolving the timing of import of foreign genes by horizontal genetic exchange, we also tested strains of *Helicobacter acinonychis,* the only close relative of *H. pylori* according to 16S RNA sequences [36,37]. The results indicate that microarrays provide useful information on variation of genome content. However, the phylogenetic history inferred by microarray data is distorted due to homoplasies, homologous recombination, and horizontal gene transfer. Given an independent phylogenetic context based on sequence diversity in core genes, these distortions can be used to elucidate the history of horizontal gene transfer into a bacterial species.

## Results

### An *H. pylori* Whole Genome Microarray

We designed a microarray from the genomes of 26695 and J99 that contains 1,649 PCR products, corresponding to 98% of the CDSs present within both genomes (Table S1). Most of the PCR products correspond to the entire CDSs, but for longer genes, only an N-terminal segment of less than 2.5 kb was amplified. Hybridizations with microarrays were measured by fluorescence with test DNAs mixed with 26695/J99 DNAs that had been differentially labeled with Cy3 and Cy5. The ratios between these fluorescence levels were categorized as reflecting presence or absence of CDSs on the basis of cut-off values that were optimized for control hybridizations between strains 26695 and J99 (accuracy: > 98%; sensitivity: 98%–100%; specificity: 82%–86% [see Materials and Methods]). Subsequent, retrospective analyses of genome content in two recently completed *Helicobacter* genomes yielded similar estimates of accuracy, sensitivity, and specificity. The estimates of specificity are probably too low: most of the apparent false-negatives seem to reflect the high efficiency of FASTA in detecting partial homologies rather than inefficient hybridization with orthologous CDSs (see Materials and Methods). Bioinformatic analyses also indicated that 98% of the PCR products on the microarray should have hybridized only with the homologous genomic region while the remainder (33/1,649) probably hybridized with two distinct regions (Table S2). Hereafter, we ignore these potential sources of error and treat positive hybridizations as indicating the presence of a CDS and negative hybridizations as indicating its absence.

### Gene Content in Representative Strains

The population structure based on the sequence diversity of seven core genes has been determined for 370 isolates from a global collection of *H. pylori* [12]; in unpublished work, this approach has been extended to more than 800 isolates (B. Linz, et al., unpublished data). We chose 56 strains of *H. pylori* to reflect the diversity within this collection and to serve as a reference strain collection (Table S3). We also tested four strains of *H. acinonychis,* a close relative of *H. pylori* that infects large felines including tigers, lions, and cheetahs [38–40]. These 60 isolates were screened against the whole gene microarrays.

**Figure 1:**
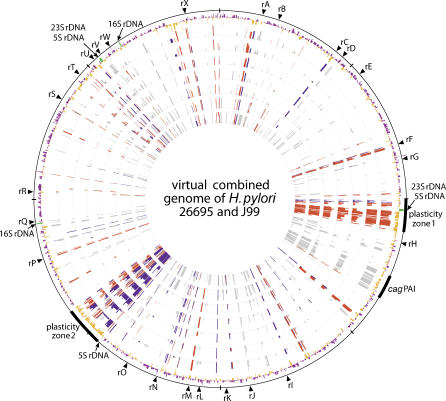
Genes Present and Absent in 56 Strains of *H. pylori* and Four Strains of *H. acinonychis* CDSs used in microarrays are shown to scale along a virtual genome consisting of CDSs from both 26695 and J99 in the gene order found within 26695. Circle contents from outside to inside: (1) virtual chromosome (1.76 Mb) with ticks every 220 kb (2), GC content indicated in colors (orange, < 39%; purple, > 39%; green, rRNA genes) (3–9), numbers of missing CDSs from individual strains according to population, color-coded according to presence in both 26695 and J99 (gray) or specific to either 26695 (red) or J99 (blue). Circle, population; 3, hpAfrica2; 4, hpAfrica1; 5, hpEurope; 6, hpAsia2; 7, hpEastAsia; 8, AmerindB; 9, *H. acinonychis*.

Microarray experiments with the 56 *H. pylori* strains showed that 499 CDSs were absent from at least one strain (Figure 1 and Table S4). As expected, genes within the *cag* PAI (28 CDSs tested) and plasticity zones 1 and 2 (108 CDSs) were lacking in numerous isolates (Figure S1), but these three regions accounted for only 27% of the 499 variably present CDSs; 73% (363 CDSs) were located in multiple regions that contained only one to eight CDSs and were scattered around the virtual genome (Figures 1 and S2). Thus, hundreds of CDSs are variably present within *H. pylori* without any obvious genomic clustering.

**Figure 2:**
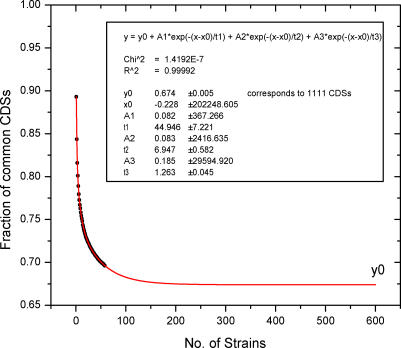
Extrapolated Number of Universally Present CDSs in *H. pylori* The fraction of CDSs present in a sample of strains (“common CDSs”) was calculated on random samples of one to 56 strains taken without replacement. Mean fractions of common CDSs were calculated from 100 iterations of this sampling procedure. The graph shows the results of fitting an exponential decay model to these calculations, in which y0 approaches the minimum number of universally common CDSs at infinity (0.674 × 1,649 CDSs = 1,111 universally present CDSs).

How many genes belong to the core genome present in all *H. pylori* strains? The two genomes contain 1,590 [21] and 1,495 [22] CDSs, respectively, for a weighted average of 1,531 CDSs (see Materials and Methods). The number of universally present CDSs decreased with the number of strains examined (Figure 2), and only 1,150 CDSs were present in all 56 strains tested (Table S5). Extrapolation to infinity indicates that the core genome consists of 1,111 CDSs (Figure 2), or 73% of the weighted average, which would be universally present in all strains even if a much larger set of isolates were tested.

### Properties of Variably Present Genes

**Figure 3:**
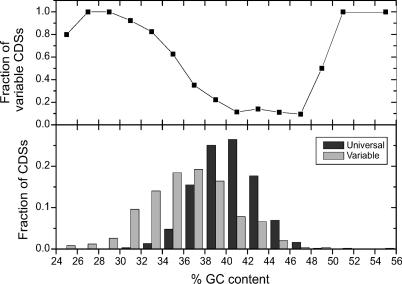
GC Content of CDSs That Are Universally Present or Variable within *H. pylori* CDSs were binned according to GC content in steps of 2% (24–26, 26–28, etc.). Top: Fraction of all CDSs within a bin that are variable. Bottom: Fraction of universally present (*n* = 1,150) or variable (*n* = 499) CDSs by GC content. One universally present CDS with a GC content of 62% (HP0359) has been excluded from the figure.

The GC content of many CDSs in the plasticity zones and within the *cag* PAI is lower than the average GC content of the entire genome [22]. An unusually low GC content is also typical of many of the variable genes outside these regions (Figure 1), but the frequency distribution of GC content among variable CDSs is unusually broad, skewing in the direction of both low and high GC content (Figure 3, bottom). In fact, most *H. pylori* CDSs with a GC content of less than 36% or more than 50% are variably present within *H. pylori* (Figure 3, top). This observation provides support for the inference that many variable genes may have been imported by horizontal gene transfer from other species [22]. The 499 variable CDSs (Table S4) have depressed the average GC content of the genome to 39%, whereas the average GC content of the 1,150 universally present genes is 40.2%.

Many of the variable genes in the plasticity region and the *cag* PAI were classified as “genes of unknown function” [22]. The same association was observed with the current dataset (Table 1): 22% of CDSs encoding outer membrane proteins, 44% of CDSs of unknown function, 54% of CDSs associated with DNA metabolism (often encoding restriction and modification enzymes), and 100% of CDSs that were assigned to “other categories,” including transposons, were variably present. Categories associated with housekeeping functions contained only a few variable CDSs (Tables 1 and S6). These observations provide additional support to the inference that many of these CDSs may have been acquired by horizontal gene transfer. Many of the same CDSs that were variable within *H. pylori* were also either lacking or variable among the four strains of *H. acinonychis* that were tested (see below).

**Table 1:**
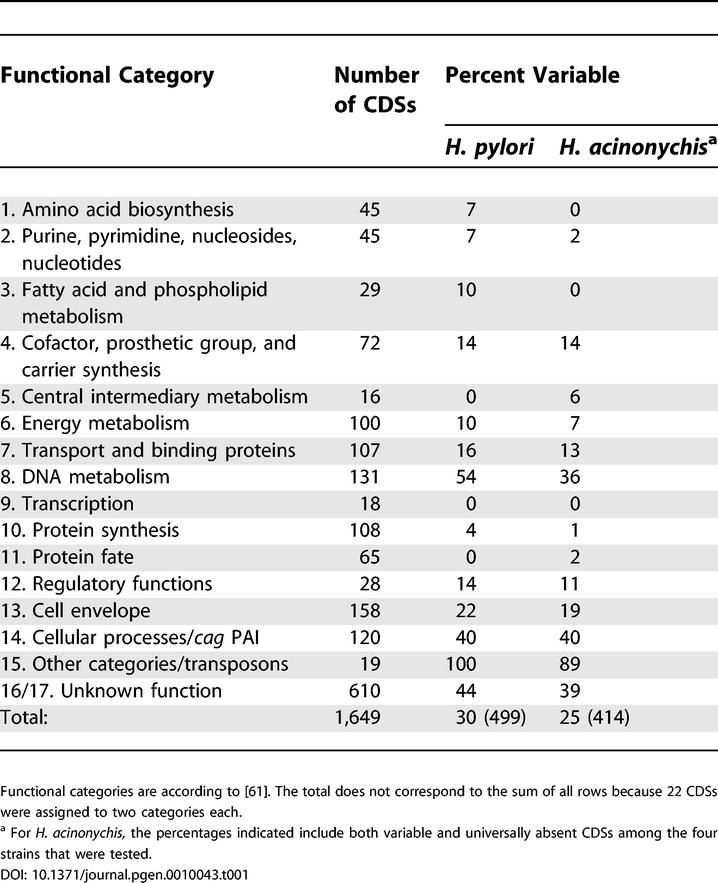
Variable CDSs in *H. pylori* and *H. acinonychis* by Functional Category

As reported by Alm et al. [22], the plasticity zone in J99 contains one of the two 5S/23S rDNA copies, and plasticity zone 2 in 26695 is flanked by an orphan 5S rDNA. Our data now show that a second 5S/23S rDNA in the genome is also associated with numerous variable CDSs, unlike the two 16S rDNA loci, where no association with variable CDSs was noted. In addition to these plasticity zones, we also noticed that the virtual genome contained 24 small regions, designated rA through rX (Figure 1), consisting of two to eight CDSs that were present in at least six isolates but largely absent in at least one population including *H. acinonychis* and AmerindB (see below).

### Differences in Genome Content by Population

Of the 499 variable CDSs, 145 (29%) were uniformly absent within at least one *H. pylori* population. These included the 28 CDSs within the *cag* PAI, 53 CDSs within plasticity zones 1 and 2 (Figure S1), 27 CDSs in regions rC, rD, rF, rM, rP, rQ, rV, rW, and rX (Figure S3), as well as 37 singleton CDSs. (However, no CDS was specific to and uniformly present in any single population, except for JHP0914, which is exclusively present in hpAfrica1.) A further indication of population structure within the microarray data was obtained by comparing the mean number of CDSs present within each population (Table 2). hpAfrica1 and hpEurope hybridized with significantly more PCR products on the microarray than did the other populations of *H. pylori,* as might be expected because the microarrays were designed using genomes from those populations (26695: hpEurope; J99: hpAfrica1). However, significant differences between the numbers of CDSs were also found in comparisons between the hpAfrica2, hpEastAsia, and hpAsia2 populations (Table 2). And *H. acinonychis* hybridized with fewer spots than did any of the *H. pylori* populations.

**Table 2:**
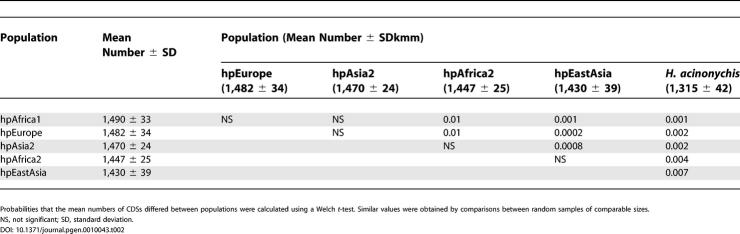
Significance of Differences in Mean Numbers of CDSs between Different Populations

**Figure 4:**
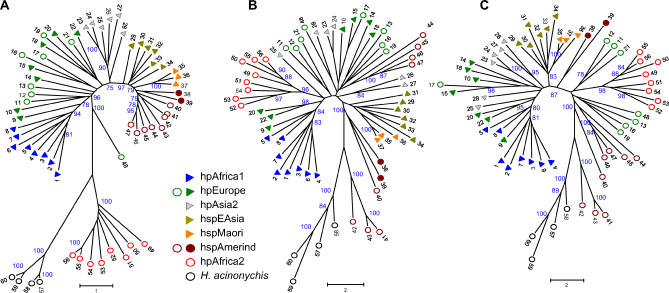
Phylogenetic Structure (Neighbor-Joining Trees) According to (A) Sequences of Seven Core Genes, (B) Microarray Data Excluding *cag* PAI, and (C) Microarray Data Including the *cag* PAI for 56 Strains of *H. pylori* and Four Strains of *H. acinonychis* Filled triangles indicate strains possessing the *cag* PAI, open circles indicate strains lacking it, and filled circles indicate hspAmerind strains that lack HP0536–0548 from the *cag* PAI. Colors indicate population assignments by Structure based on the sequence data (B. Linz, unpublished data). Numbers at the tips of the twigs are strain numbers (Table S3), while blue numbers next to nodes are bootstrap values over 75% after 250 iterations.

In order to test the strength of these correlations, we constructed pair-wise difference matrices for all 60 strains based on the nucleotide content for all seven core genes and on hybridization with the microarray. These matrices were used to calculate Neighbor-joining phylogenetic trees whose branch order was compared by Pearson correlation coefficients *(r).* The phylogenetic trees differed considerably in branch order (Figure 4A versus 4C; *r* = 0.49). Many of the discrepancies seem to reflect the 28 CDSs in the *cag* PAI, because *cag* ^+^ isolates clustered separately from *cag^−^* isolates (Figure 4C), unlike the relationships according to the seven core genes. In microarray trees within which the CDSs within the *cag* PAI were excluded (Figure 4B), *cag* ^+^ and *cag^−^* isolates of hpEurope were intermingled, and isolates from hpAfrica1, hpAfrica2, hspEAsia, hspMaori, and *H. acinonychis* formed distinct clusters, similar to the sequence tree (Figure 4A). However, this tree still differed considerably from the sequence-based tree (*r* = 0.51). In the microarray tree, hpEurope and hpAsia2 were intermingled, the hspAmerind population was split into two distinct groupings, and hpAfrica2 was more closely related to hpEurope than according to sequence data, where it formed a highly distinct branch. Finally, the sequence tree is tripartite, consisting of *H. acinonychis,* hpAfrica2, and a continuum of strains from the four other populations. In contrast, according to the microarray data, *H. acinonychis* is most similar to three strains (strains 41–43) of the hspAmerind subpopulation that were isolated from Athabaskans in Canada and which we shall subsequently refer to as AmerindB. We note that hpAfrica2 and hspAmerind were identified only due to considerable efforts to cover the global phylogeographical diversity of *H. pylori* [12]. We therefore tested whether a less globally representative population sample would have yielded stronger correlations between microarrays and sequences of core genes. After excluding the hpAfrica2 population and hspAmerind isolates, the matrices were indeed much more similar (*r* = 0.85, regardless of whether or not the *cag* PAI was excluded).

### *cag* PAI Association with Population

The entire *cag* PAI was lacking in the four isolates of *H. acinonychis* and eight isolates of hpAfrica2 that were tested by microarrays (Figure S1), which sequence experiments showed was due to the presence of an empty site. Other experiments using PCR amplification also demonstrated an empty site at the location of the *cag* PAI in five additional isolates of *H. acinonychis* and ten additional isolates of hpAfrica2 (unpublished data). All isolates of the populations hpAfrica1 and hpAsia2, as well as the subpopulations hspEAsia and hspMaori, contained the entire *cag* PAI. In contrast, some isolates of hpEurope contained the *cag* PAI while others lacked it. Similarly, most hspAmerind isolates lacked the *cag* PAI while others possessed only part of the *cag* PAI and were lacking CDSs between HP0536 and HP0548. The AmerindB group all lacked the entire *cag* PAI.

### Similarities between *H. acinonychis* and AmerindB

*H. acinonychis* and AmerindB are not particularly closely related on the basis of nucleotide sequences. The observation that they cluster near each other according to the microarray data (Figures 4B, 4C, and S3) was intriguing as it indicates that multiple CDSs are absent in both groups, possibly due to convergent evolution. From the microarray, 243 CDSs were universally absent in the four isolates of *H. acinonychis* and 170 others were variably present. Within the three isolates of AmerindB, 223 CDSs were absent and 110 were variable. These sample sizes are very small, and it may be more relevant to compare with CDSs that were either absent or variably present. Of the 413 such CDSs in *H. acinonychis* and 333 in AmerindB, the great majority (288), including 83 in the plasticity zones and 54 in regions rA to rX, were variable or absent in both groups. These included the *cag* PAI (Figure S1) as well as 181 CDSs of unknown function. Interestingly, of those CDSs for which functional assignments had been made, eight were associated with molybdenum, namely HP0172 and HP0798–0801 (region rK), which are involved in molybdopterin biosynthesis, as well as HP0473–0475 (region rH), which encode a molybdenum ABC transporter. These eight CDSs were generally present in other strains, including other hspAmerind isolates (Table S4). Similarly, ten outer membrane protein genes (HP0009, 78, 79, 252, 492, 722, 725, 922, 1243, and 1453) were absent in these two groups, as were four restriction or modification genes. One possible explanation for these observations is that some of the CDSs lacking in both groups reflect adaptation to a similar ecological niche of currently unknown nature.

Other CDSs were specific for only one of the two groups: *H. acinonychis* lacks CDSs encoding six outer membrane proteins and 17 hypothetical proteins that are present in AmerindB, whereas AmerindB lacks 12 CDSs encoding proteins with various functions that are present in *H. acinonychis* (Table S4). AmerindB strains also lack *amiE* (HP0294) and *amiF* (HP1238), encoding aliphatic amidases that are implicated in ammonia production. All other strains that were tested harbor both CDSs, except for two *H. acinonychis* isolates that possess only *amiE*. The absence of these CDSs was unexpected because ammonia production was thought to be very important for colonization of the stomach by *Helicobacter* [41].

## Discussion

Microarrays containing 1,649 CDSs that are present in the genomes of 26695 and J99 were tested against a representative sample of the population genetic diversity within a global collection of *H. pylori* and *H. acinonychis*. After weighting for CDSs that are specific to each of the two genomes, 25% of the CDSs within these two genomes were absent in one or more of 56 isolates of *H. pylori,* and 21% of the *H. pylori* CDSs were absent or variable within four isolates of *H. acinonychis*. After extrapolation, we infer that the core genome that is universally present within all strains of *H. pylori* consists of only 1,111 CDSs (73% of the weighted average) (Figure 2).

### Variable Genes: Loss or Gain?

Does the variable presence of CDSs reflect acquisition by some isolates, or loss by others, or a combination of both processes? The sequence-based tree in Figure 4A provides a framework for addressing this question because it reflects the evolutionary history of *H. pylori* relative to its closest relative, *H. acinonychis* [36]. This tree is tripartite and consists of three lobes: *H. acinonychis,* hpAfrica2, and a near continuum of isolates from hpEastAsia, hpAsia2, hpEurope, and hpAfrica1. Sequence comparisons with other *Helicobacter* species place the root of this tree near the branch between hpAfrica2 and *H. acinonychis* (unpublished data). Therefore, CDSs that are present within the other populations, but absent in hpAfrica2 and *H. acinonychis* (and other *Helicobacter* species), are likely to have been acquired after speciation by horizontal gene transfer from unrelated species. Once imported, such CDSs can spread between isolates by DNA transformation and homologous recombination at the flanking sequences. Alternatively, spread of an empty site by transformation [9] can also lead to loss of such sequences.

Based on this reasoning, the *cag* PAI was probably imported by horizontal gene transfer from a different species after the tripartite split within the tree because neither *H. acinonychis* nor the hpAfrica2 population (nor the distant species *Helicobacter hepaticus* [42] or *Wolinella succinogenes* [43]) possess any of the CDSs within the *cag* PAI. The *cag* PAI is present in almost all hpAfrica1, hpEastAsia, and hpAsia2 isolates and many hpEurope isolates. However, it is lacking completely or in part in most hspAmerind isolates, which also belong to hpEastAsia, and half of the hpEurope isolates. One possibility to explain these observations is that selection for the type-four secretion system encoded by the *cag* PAI [44–46] may have resulted in the descent of these four populations from an ancestor that had already imported the *cag* PAI. In that case, strains lacking the *cag* PAI, or parts of it, have lost the island through transformation with an empty site or through deletion mutations. Alternatively, the *cag* PAI was imported after subdivision into the hpAfrica1, hpAsia2, hpEastAsia, and hpEurope populations. Its current presence in all of these populations would then reflect spread by transformation from the cells that had first acquired it, coupled with selection for its expression. Extant isolates lacking the *cag* PAI would then represent the ancestral state prior to its acquisition, or in some cases secondary loss due to transformation of an empty site. Both alternative scenarios infer that *H. pylori* containing the *cag* PAI are fitter than those lacking it and that the high number of strains carrying this island reflects positive selection for *cag* ^+^ strains. However, the first alternative infers that the selection pressures may not be very high. Although *H. pylori* possessing the *cag* PAI are more virulent than strains lacking it [2], both *cag* ^+^ and *cag* ^−^ strains are of comparable incidence within Spain [47], and it remains unclear whether *cag* ^+^ bacteria are any fitter in terms of transmission than are *cag* ^−^ bacteria. Weak support for the first alternative is also provided by the consideration that transformation of the *cag* PAI may be very rare due to its size (38 kb); the median size of DNA fragments that are exchanged by recombination is only 450 bp [10]. Further sequence analyses of the regions flanking the *cag* PAI would be needed to distinguish between these alternative scenarios

### Other Variably Present CDSs

The situation for most of the other variable CDSs differs from that of the *cag* PAI. These CDSs tend to be short (Figure S4) and are located within multiple regions, many of which contain fewer than eight CDSs (Figure 1). Thus, they should be readily transmissible between strains by homologous recombination at flanking sites after DNA transformation. Like the *cag* PAI, these CDSs might have represented genes that were imported recently and have spread due to selection. However, most of the variable CDSs were found in many or all of the *H. pylori* populations, including hpAfrica2, indicating that they were probably inherited from the last common ancestor of this species. If they were imported from an unrelated species, this must then have happened prior to the existence of that last common ancestor. Their low GC content (Figure 3) is not incompatible with this interpretation, because amelioration of GC content is a process that can take millions of years [48]. Furthermore, CDSs that are absent within certain groups of isolates, such as the AmerindB group plus *H. acinonychis* (Figure S2)*,* probably reflect convergent, independent gene loss, because the populations lacking these genes are not particularly closely related. Similar conclusions have been drawn for variably present CDSs within the Enterobacteriaceae that were imported once and have subsequently been lost on multiple occasions during subsequent evolution [49].

Most of the variable CDSs encode proteins of unknown function, or selfish DNA such as restriction or modification enzymes [50], and may not possess functional attributes that are targets for positive selection. Thus, it seems likely that repeated loss rather than recent acquisition accounts for the variability of so much of the *H. pylori* genome. Note that if variable CDSs were imported after speciation, their presence in multiple populations would reflect spread by transformation and would result in less geographical diversity than is the case for neutral, housekeeping genes, whose genetic diversity reflects genetic drift associated with geographical separation. Sequence comparisons of such genes could test this interpretation.

### Evolutionary Analyses with Microarray Data

A major presumption within our analyses is that the population structure revealed by sequencing housekeeping genes is a more accurate representation of the genetic descent than are the relationships revealed by microarray analyses. We also infer that this may be true for bacteria in general. For *Y*. *pestis,* a species with only limited diversity, microarray analysis involving 4,000 CDSs [29,32] was concordant with the branch order revealed by 44 synonymous single nucleotide polymorphisms [28], but less informative. Within *S. enterica,* microarrays based on 4,300 CDSs yielded trees that were generally concordant with those based on multilocus enzyme electrophoresis using 24 enzymes [34], but multiple exceptions were found. Our observations with *H. pylori* parallel those with other species, namely that there is general concordance between phylogenetic relationships based on a microarray with 1,649 CDSs and sequences from seven gene fragments. However, multiple discrepancies were found and the two methods yielded distinct patterns of relationship. The Pearson correlation coefficient between dissimilarity matrices from microarrays and sequence data for the same 60 isolates was only 0.5. Microarrays grouped organisms together (AmerindB and *H. acinonychis*) that belong to different species and are not known to have any particularly close evolutionary relationship. As a result, the microarray data did not detect the tripartite population structure within these isolates that is revealed by sequence analysis. It might be argued that microarrays based on all the CDSs within a genome provide a better overview of relationships than do the sequences of a few core genes. We note that at least for *H. pylori,* this is probably not the case because the information content in the sequences of seven gene fragments (1,480 polymorphic sites can yield 4^1,480^ distinct combinations), is 730 orders of magnitude greater than the presence or absence of 535 CDSs (2^535^ distinct combinations). Secondly, hybridization data from microarrays have a certain methodologically inherent inaccuracy, unlike sequence data. Furthermore, established phylogenetic methods and theory are available for evaluating sequence differences, including the ability to calculate population structures and the time since the existence of a last common ancestor. Until now, microarray data have been evaluated predominantly by clustering techniques, and it is unclear whether changes occur according to a molecular clock or not. In addition, high-throughput methods allow sequencing of multiple gene fragments from thousands of isolates (http://www.mlst.net), which can be necessary for population studies, whereas microarray analysis of more than 100 isolates remains a major effort. Finally, microarrays are based on the gene complement of the genomes that have been chosen for sequencing, which can provide a biased view of the global diversity of that particular species [51]. It therefore seems most appropriate to continue to use sequences of multiple core genes for determining population structure.

The data presented here also show that the comprehensive overview of the genomic content of multiple isolates from microarray data can be used in the context of a known population structure in order to identify discrepancies that reflect evolution by gene acquisition and loss rather than by descent. Such discrepancies can then be used to infer when genes were acquired (or lost) and to infer the selective advantages of particular genes on a genome-wide scale. We therefore conclude that the power of genome-wide analyses of microarrays is first released when analyzed in the context of a population structure that has been defined by sequence based methods.

### Conclusions

The data presented here provide a rich source of information on variability within *H. pylori* and *H. acinonychis*. Unlike previous conclusions [22], the genome of these organisms is plastic and a weighted average of 27% of the genome is variably present in different isolates. Our data provide a phylogenetic hypothesis for when the *cag* PAI and other variable regions were imported into these species and indicate that convergent evolution has occurred within the AmerindB group and *H. acinonychis*. In addition, they also provide a list of 1,150 core genes (Table S5), most of which are universally present within *H. pylori,* and which includes genes that are essential for the unique physiology of this organism.

## Materials and Methods

### Bacterial isolates.

*H. pylori* strains, numbered 1 to 56, that are representative of the hspWAfrica (*n* = 3) and hspSAfrica (*n* = 5) subpopulations of hpAfrica1, hpAfrica2 (*n* = 8), hpEurope (*n* = 15), hpAsia2 (*n* = 6), and the hspEAsia (*n* = 6), hspMaori (*n* = 3), and hspAmerind (*n* = 10) subpopulations of hpEastAsia were investigated. Four *H. acinonychis* strains, numbered 57–60, were from a cheetah, a lion, and two tigers from the United States and Russia. Details about these bacterial strains are summarized in Table S3. Bacterial strains were cultivated as previously described [45]. Genomic DNA was isolated using Qiagen Genomic DNA isolation kits according to the manufacturer's instructions (Qiagen, Valencia, California, United States).

### Housekeeping genes.

Fragments of the housekeeping genes *atpA, efp, mutY, ppa, trpC, ureI,* and *yphC* were amplified, and both strands were sequenced from independent PCR products as described [18].

### Microarray experiments.

The *H. pylori* array consisted of 1,649 PCR fragments corresponding to 98% of the CDSs within the genomes of 26695 and J99 [21,22]. Primers were designed manually that amplified each entire CDS, or, for long CDSs, the N-terminal 2 kb, with flanking universal linkers to allow reamplification. PCR products were amplified from genomic DNA of 26695 (1,558 CDSs), except for 91 CDSs that were amplified from J99, to which they are specific. After dilution, these PCR products were then used as templates for three secondary rounds of amplification with primers specific for the universal linkers. For each PCR product, agarose gel electrophoresis was used to confirm that single bands of the correct size had been amplified. For PCR products that did not meet this criterion, novel primers flanked by universal linkers were designed using PrimeArray [52]. The final list of primers and sizes of amplified genomic DNA within the PCR products (size range: 62–2,568 bp) is presented in Table S1. Upon bioinformatic analysis of the information in Table S1, we noticed that the PCR product for JHP1032 should not correspond to that CDS, because the primers are oriented in the wrong direction. All other primers were confirmed by FASTA analyses to correspond to the sequence positions listed in Table S1. The same microarray has been used previously for transcriptional analyses [53].

PCR products from the secondary round of amplification were purified using Millipore MultiScreen-PCR plates( Millipore, Billerica, Massachusetts, United States), resuspended in printing buffer (150 mM sodium phosphate [pH 8.5], 0.01% N-lauroyl sarcosine [Sigma, St. Louis, Missouri, United States]) and spotted in duplicate with a Microgrid II spotter (Biorobotics; Genomic Solutions, Ann Arbor, Michigan, United States) on glass slides coated with poly-L-lysine (http://cmgm.stanford.edu/pbrown/protocols/1_slides.html). Additional negative controls were also spotted onto the slides, consisting of 20 PCR fragments from human IMAGE clones from the I.M.A.G.E. Consortium (http://image.llnl.gov/), printing buffer or water. Bacterial genomic DNA was fluorescently labeled with either Cy3 or Cy5 during three rounds of extension with Klenow enzyme and purified as described (http://cmgm.stanford.edu/pbrown/protocols/4_genomic.html). 2 μg of purified labeled DNA from each test strain was mixed with 1 μg each from 26695 and J99 that had been labeled with the alternate dye and hybridized in DIG Easy Hyb buffer (Roche, Basel, Switzerland) to a microarray slide for 15–18 h at 37 °C. DIG Easy Hyb buffer contains urea in a concentration that results in hybridization conditions that are comparable to 50% formamide content. The arrays were washed in washing buffer 1 (1×SSC, 0.03% SDS) until the cover slip fell off, followed by 10-min washes with washing buffers 2 (0.2×SSC) and 3 (0.05×SSC) and dried under a stream of gaseous nitrogen. Fluorescent signal intensities were measured with a G2565AA scanner (Agilent Technologies, Palo Alto, California, United States), and feature extraction was performed with ImaGene (Biodiscovery, El Segundo, California, United States). Empty spots and spots with impurities or high backgrounds were flagged and excluded from the analysis (“missing data”). Spots with signal intensities below the average cross-hybridization signal of the IMAGE clone spots were also excluded. Local background values were subtracted from each spot, the intensities for each fluorescent signal were normalized to the mean intensities over the entire microarray, and the signal intensity ratios between the two dyes were calculated for each PCR product.

The data presented here are based on mean ratios of signal intensities from two experiments each with dye-swapping. For half of the experiments, multiple spots were excluded due to high backgrounds and one to two additional slides were therefore tested. The mean ratios were based on the data from all 2–4 slides that were tested, except when more than two slides were tested, in which case unusually high or low single values were excluded from the mean ratios. The mean ratios were log_2_ transformed and were assigned a 0 or 1 on the basis of an optimized cut-off calculated by using Gack [54] (settings: data smoothing by moving window average 7; peak modeling based on the normal curve; binary output with 25% EPP cut-off). These settings were determined by trial and error to yield the greatest accuracy (maximal percentage of correct assignments) in separate, control hybridizations between 26695 and J99 (J99: 97%; 26695: 99%) on the basis of the original assignments of genome specificity [22]. We estimated the number of false-positives (J99, 24/135 (18%); 26695, 12/91 (13%) and false-negatives (J99, 25/1514 (1.7%); 26695: 0/1558) and calculated sensitivity (J99: 98%; 26695: 100%) and specificity (J99: 82%; 26695: 86%) values as described in Table S7. We note that 67 of these original assignments to genome-specific genes were predicted to yield false-positive results by Salama et al. [24] on the basis of bioinformatic analyses whereas still other genes are predicted to yield apparently false assignments by Kim et al. [54].

Two sets of retrospective bioinformatic analyses were performed to determine the specificities of the assignments. Firstly, we identified the best FASTA [55] hits for each PCR product included on the array within two unpublished genome sequences (*H. acinonychis* strain Sheeba [from S. C. Schuster] and hpAfrica2 strain 162.0 [from SS and MA] of isolates that had been tested by microarrays). For each best hit, we calculated a measure of similarity, zfp, consisting of the normalized Z-score [56] multiplied by the homology, multiplied by the fractional length of the hit compared with the size of the PCR product. Most PCR products that hybridized with the microarrays possessed high zfp values for both genomes (Figure S5, bottom), but the distribution of zfp values for PCR products that did not hybridize was very broad (Figure S5, top), and overlapped in part with the distribution of the positive results. The validity of using zfp as a predictor for hybridization was investigated by visual examination of genomic comparisons in ACT [57], resulting in the assignments summarized as + and − for predicted hybridization in Figure S5. According to these manual analyses, most PCR products with zfp > 91 had high homologies over extensive stretches to a genomic region and might be expected to result in positive hybridizations while most PCR products with lower zfp values had only poor homologies to short stretches. Based on a cut-off value of 91, we calculated the accuracy as 93%–96%, sensitivity as 96%–98%, and specificity as 80%–83% (Table S7). These are reflected by zfp values below 91 in 2.1% (Sheba: 28/1314) to 2.5% (162.0: 36/1453) of positive hybridizations and zfp values > 91 in 13.7% (Sheba; 43/315) to 14.6% (162.0; 26/178) of negative hybridizations. However, zfp may not be fully adequate to predict hybridization because 79% (Sheba) to 81% (162.0) of the hits for apparent false-negatives (zfp > 91; hybridization-positive) possessed either < 83% homology or were homologous to a stretch of < 250 bp. As a result, the calculated sensitivity and specificity values are probably much too conservative.

In the second approach, we used FASTA analysis to identify PCR products that would hybridize in microarrays with other sequences within the genome rather than that of the CDS for which the primers had been designed. To this end, we identified all FASTA hits with zfp values > 91 that were not within the location targeted by the PCR primers. We predict that 2% (33/1649) of the PCR products should each hybridize detectably with a second location (Table S2).

### Data analysis.

A virtual genome was calculated in which J99 CDSs plus specific flanking DNA were inserted at the appropriate positions based on flanking CDSs within the 26695 genome, and printed using a modified version (http://www.uniklinikum-giessen.de/genome/) of GenomeViz [58] (Figures 1 and S2). The number of CDSs lacking from this virtual genome was calculated for individual strains after weighting CDSs that were specific for 26695 (134 CDSs, weighting factor of 0.4) or J99 (91 CDSs, 0.6).

Functional annotations of genes are according to http://genolist.pasteur.fr/PyloriGene/. 1,627 genes were assigned to a single functional category, and 22 were assigned to two categories.

Statistical analyses were performed using functions that are implemented in R [59]. The statistical significance of differences between mean numbers of CDSs per population in Table 2 was calculated using the “*t*-test” function for all the data and for random samples of comparable size (“sample” function) over 100 iterations.

Neighbor-joining trees were calculated from difference matrices based on Hamming distances with Mega V2.0 [60]. The correlations between these trees were calculated by Mantel tests with GENETIX (http://www.univ-montp2.fr/~genetix/genetix/intro.htm).

## Supporting Information

Figure S1 CDSs Present or Absent within the *cag* PAI and the Plasticity Zones among 56 Strains of *H. pylori* and Four Strains of *H. acinonychis*
Red, absent; yellow, present; black, missing data.
(1 MB PDF)

Figure S2 A Higher-Resolution Version of Figure 1
(313 KB PDF)

Figure S3 CDSs Present or Absent within 24 Small Regions of 56 *H. pylori* and Four *H. acinonychis* Strains These regions are scattered along the chromosome, contain two to nine CDSs and contain CDSs that are uniformly lacking in at least one population. Red, absent; yellow, present; black, missing data.
(1 MB PDF)

Figure S4 Size Distribution of Variably or Universally Present CDSs within *H. pylori* and *H. acinonychis*
(10 KB PDF)

Figure S5 Bioinformatic Predictions of Hybridization based on FASTA Analyses of the Genomic Sequences of hpAfrica2 Strain 162.0 and *H. acinonychis* strain Sheeba PCR products on the microarray that hybridized with each of these strains are shown in the lower quadrants, while those that did not hybridize are shown in the upper quadrants. Each quadrant shows a histogram of the number of PCR products versus zfp from FASTA searches (bottom) and whether individual PCR products were predicted to hybridize on the basis of manual ACT comparisons (top). zfp was calculated as the normalized Z-score × homology × fractional length of hit. The arrow in the top left quadrant shows the position of zfp = 91, which was used as a cut-off value to distinguish between FASTA hits that would be expected to hybridize based on the ACT comparisons. These data form the basis for the calculations in Table S7.
(50 KB PDF)

Table S1 List of PCR Products (152 KB TXT)

Table S2 List of PCR Products with Multiple Hits within the Genome (3 KB TXT)

Table S3 List of Strains (27 KB XLS)

Table S4 List of 499 Variable CDSs in *H. pylori*
(287 KB XLS)

Table S5 List of 1,150 PCR Products that Hybridized with All *H. pylori* Strains (14 KB TXT)

Table S6 Variable CDSs in *H. pylori* (sub)populations (7 KB PDF)

Table S7 Accuracy, Sensitivity, Specificity and Predictive Value of Hybridizations with the Genomic Sequences of Sheba and 162.0 (47 KB PDF)

## Abbreviations

*cag* PAI: *cag* pathogenicity island
CDS: coding sequence
kb: kilobase
