# Supplementary material for: Gain and Loss of Multiple Genes During the Evolution of Helicobacter pylori
Source: PLoS Genet. 2005 Oct 7;1(4):e43. doi: 10.1371/journal.pgen.0010043 (PMC1245399; doi:10.1371/journal.pgen.0010043)
Supplement: Supplementary file 1 [file pgen.0010043.sg001.pdf]

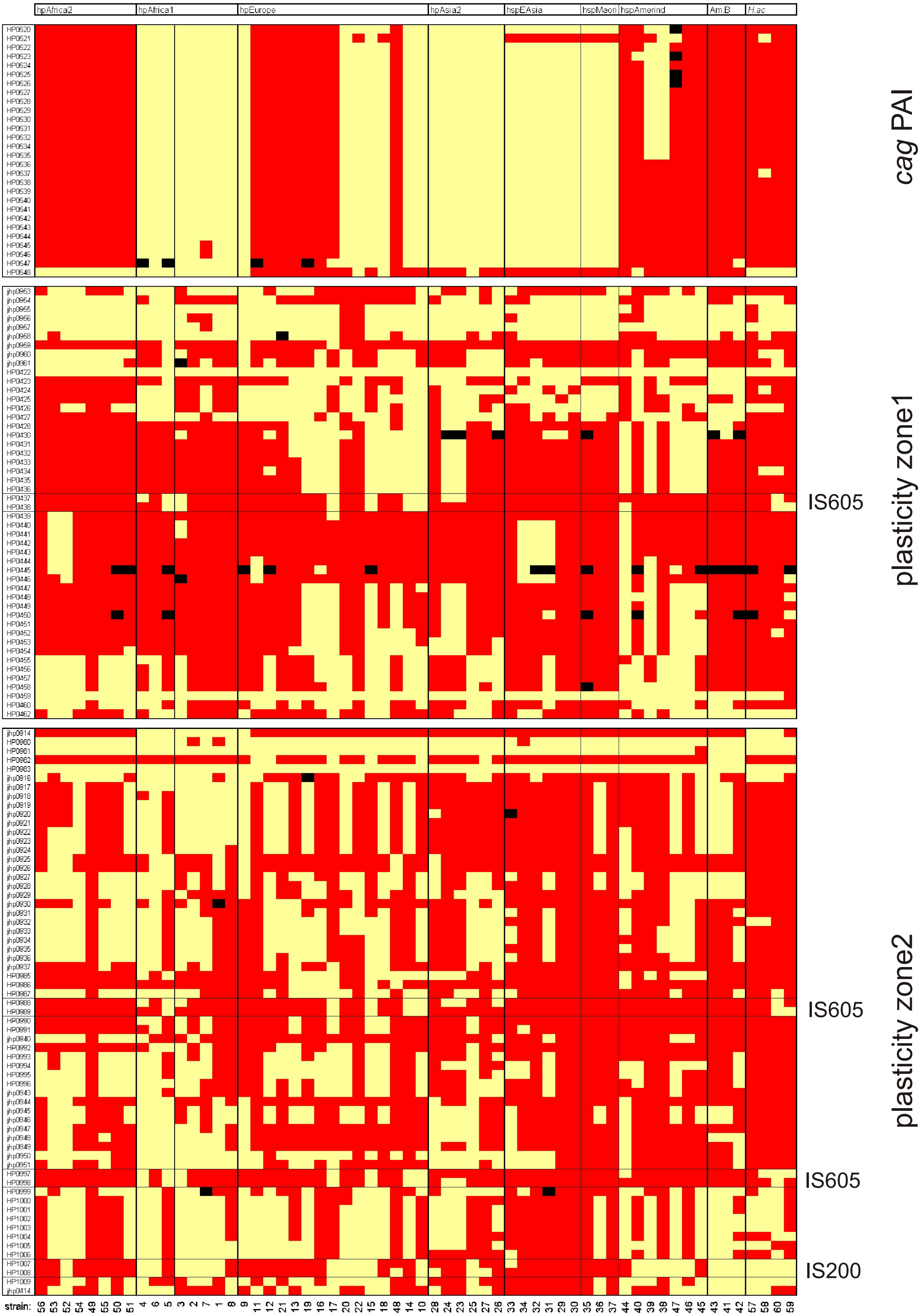

hpAfrica2
hpAfrica1
hpEurope
hpAsia2
hspEAsia
hspMaori
hspAmerind
Am.B
H.ac
HP0520
HP0521
HP0522
HP0523
HP0524
HP0525
HP0526
HP0527
HP0528
HP0529
HP0530
HP0531
HP0532
HP0534
HP0535
HP0536
HP0537
HP0538
HP0539
HP0540
HP0541
HP0542
HP0543
HP0544
HP0545
HP0546
HP0547
HP0548
cag PAI
jhp0953
jhp0954
jhp0955
jhp0956
jhp0957
jhp0958
jhp0959
jhp0960
jhp0961
HP0422
HP0423
HP0424
HP0425
HP0426
HP0427
HP0428
HP0430
HP0431
HP0432
HP0433
HP0434
HP0435
HP0436
HP0437
HP0438
HP0439
HP0440
HP0441
HP0442
HP0443
HP0444
HP0445
HP0446
HP0447
HP0448
HP0449
HP0450
HP0451
HP0452
HP0453
HP0454
HP0455
HP0456
HP0457
HP0458
HP0459
HP0460
HP0462
IS605
plasticity zone1
jhp0914
HP0980
HP0981
HP0982
HP0983
jhp0916
jhp0917
jhp0918
jhp0919
jhp0920
jhp0921
jhp0922
jhp0923
jhp0924
jhp0925
jhp0926
jhp0927
jhp0928
jhp0929
jhp0930
jhp0931
jhp0932
jhp0933
jhp0934
jhp0935
jhp0936
jhp0937
HP0985
HP0986
HP0987
HP0988
HP0989
HP0990
HP0991
jhp0940
HP0992
HP0993
HP0994
HP0995
HP0996
jhp0943
jhp0944
jhp0945
jhp0946
jhp0947
jhp0948
jhp0949
jhp0950
jhp0951
HP0997
HP0998
HP0999
HP1000
HP1001
HP1002
HP1003
HP1004
HP1005
HP1006
HP1007
HP1008
HP1009
jhp0414
IS605
IS605
IS200
plasticity zone2
strain:
56 53 52 54 49 55 50 51 4 6 5 3 2 7 1 8 9 11 12 21 13 19 16 17 20 22 15 18 48 14 10 28 24 23 25 27 26 33 34 32 31 29 30 35 36 37 44 40 39 38 47 46 45 43 41 42 57 58 60 59
