# Supplementary material for: Gain and Loss of Multiple Genes During the Evolution of Helicobacter pylori
Source: PLoS Genet. 2005 Oct 7;1(4):e43. doi: 10.1371/journal.pgen.0010043 (PMC1245399; doi:10.1371/journal.pgen.0010043)
Supplement: Supplementary file 2 [file pgen.0010043.sg002.pdf]

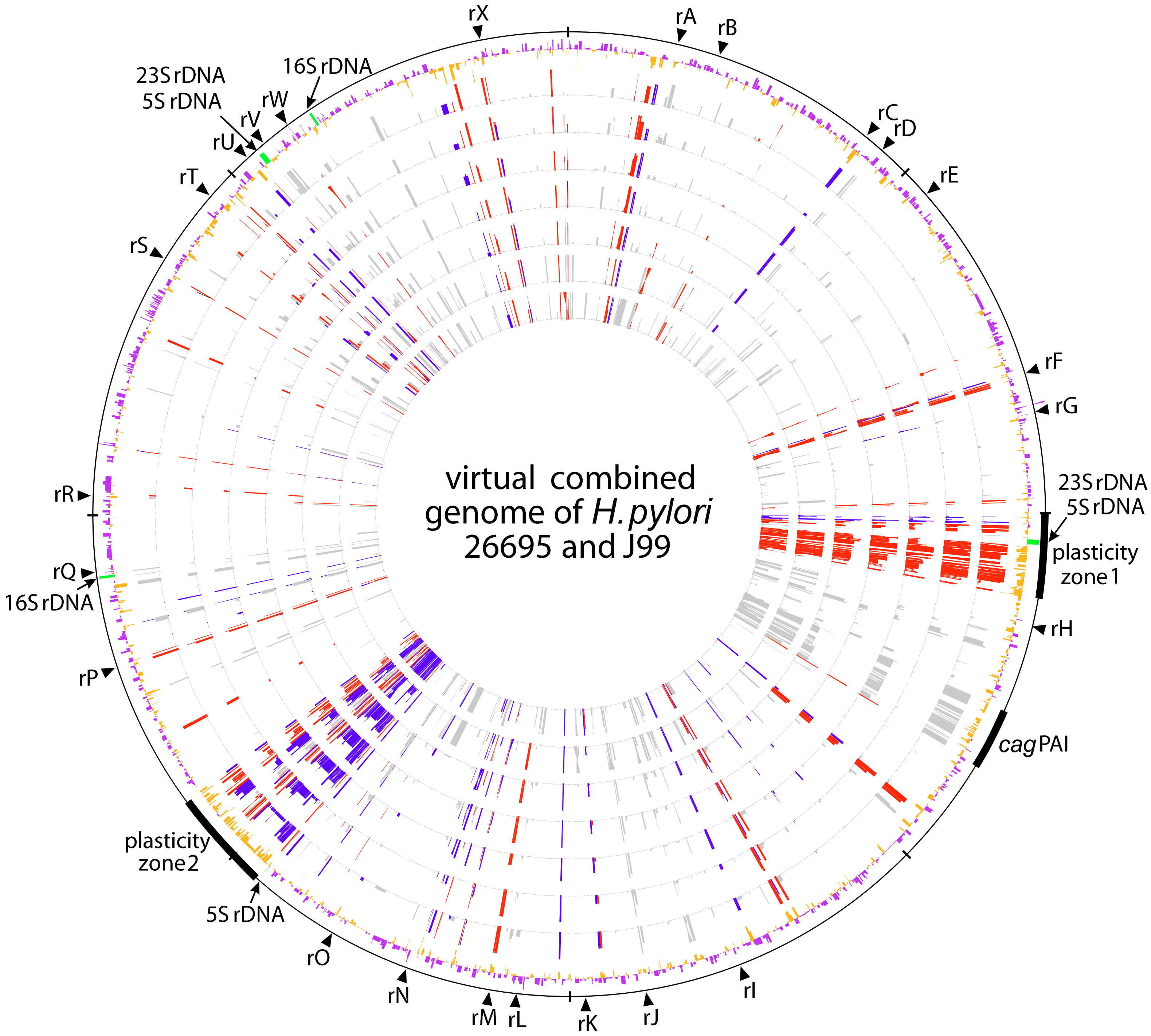

virtual combined
genome of H. pylori
26695 and J99
rX
rA
rB
rC
rD
rE
rF
rG
23S rDNA
5S rDNA
plasticity
zone 1
rH
cag PAI
rI
rJ
rK
rL
rM
rN
rO
5S rDNA
plasticity
zone 2
rP
16S rDNA
rQ
rR
rS
rT
rU
rV
rW
23S rDNA
5S rDNA
16S rDNA
