# Supplementary material for: Gain and Loss of Multiple Genes During the Evolution of Helicobacter pylori
Source: PLoS Genet. 2005 Oct 7;1(4):e43. doi: 10.1371/journal.pgen.0010043 (PMC1245399; doi:10.1371/journal.pgen.0010043)
Supplement: Supplementary file 3 [file pgen.0010043.sg003.pdf]

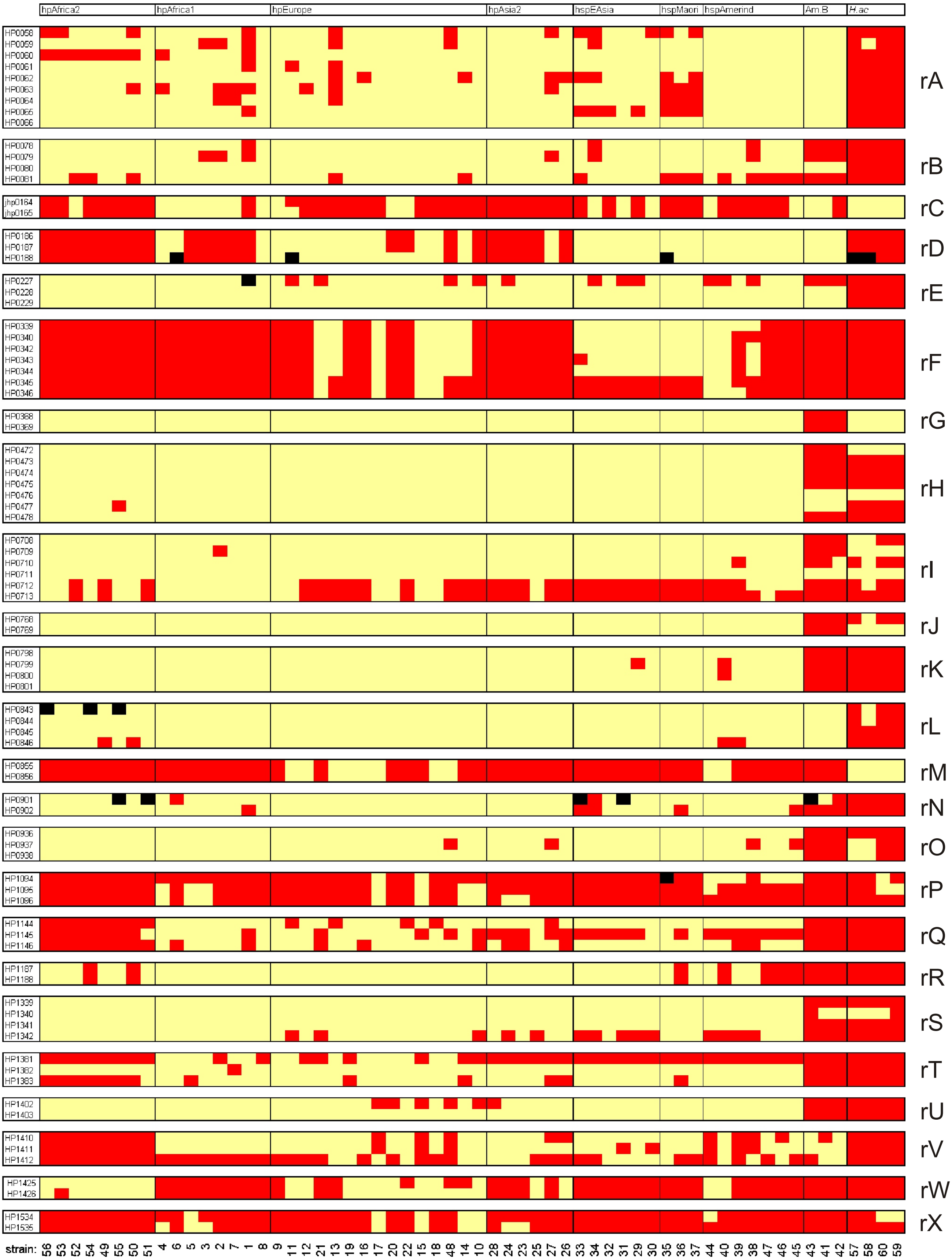
hpAfrica2
hpAfrica1
hpEurope
hpAsia2
hspEAsia
hspMaori
hspAmerind
Am.B
H.ac
HP0058 HP0059 HP0060 HP0061 HP0062 HP0063 HP0064 HP0065 HP0066
rA
HP0078 HP0079 HP0080 HP0081
rB
jhp0164 jhp0165
rC
HP0186 HP0187 HP0188
rD
HP0227 HP0228 HP0229
rE
HP0339 HP0340 HP0342 HP0343 HP0344 HP0345 HP0346
rF
HP0368 HP0369
rG
HP0472 HP0473 HP0474 HP0475 HP0476 HP0477 HP0478
rH
HP0708 HP0709 HP0710 HP0711 HP0712 HP0713
rI
HP0768 HP0769
rJ
HP0798 HP0799 HP0800 HP0801
rK
HP0843 HP0844 HP0845 HP0846
rL
HP0855 HP0856
rM
HP0901 HP0902
rN
HP0936 HP0937 HP0938
rO
HP1094 HP1095 HP1096
rP
HP1144 HP1145 HP1146
rQ
HP1187 HP1188
rR
HP1339 HP1340 HP1341 HP1342
rS
HP1381 HP1382 HP1383
rT
HP1402 HP1403
rU
HP1410 HP1411 HP1412
rV
HP1425 HP1426
rW
HP1534 HP1535
rX
strain: 56 53 52 54 49 55 50 51 4 6 5 3 2 7 1 8 9 11 12 21 13 19 16 17 20 22 15 18 48 14 10 28 24 23 25 27 26 33 34 32 31 29 30 35 36 37 44 40 39 38 47 46 45 43 41 42 57 58 60 59
