# Supplementary material for: Gain and Loss of Multiple Genes During the Evolution of Helicobacter pylori
Source: PLoS Genet. 2005 Oct 7;1(4):e43. doi: 10.1371/journal.pgen.0010043 (PMC1245399; doi:10.1371/journal.pgen.0010043)
Supplement: Supplementary file 4 [file pgen.0010043.sg004.pdf]

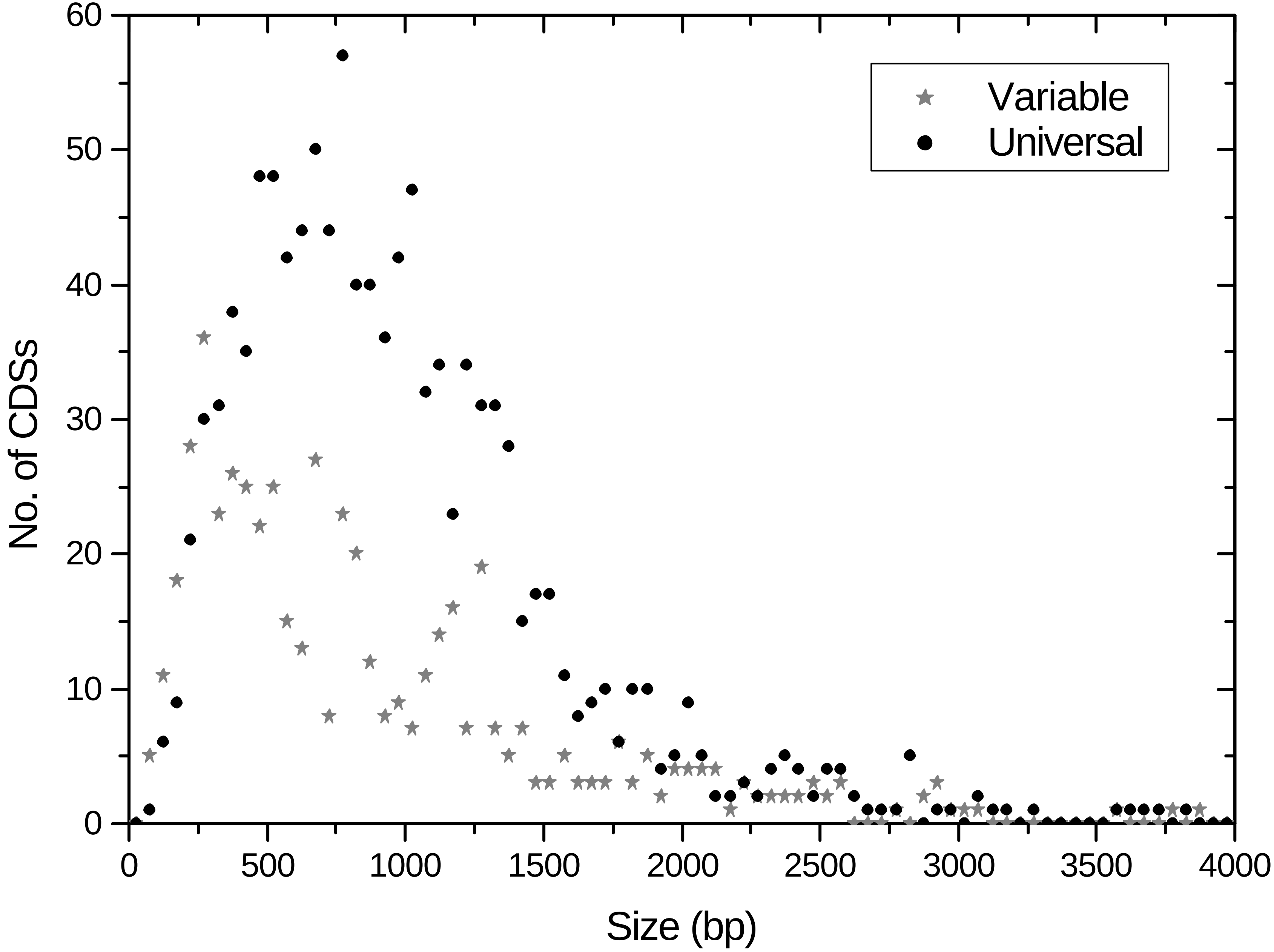

Variable
Universal
No. of CDSs
Size (bp)
0
10
20
30
40
50
60
0
500
1000
1500
2000
2500
3000
3500
4000
