# Supplementary material for: Gain and Loss of Multiple Genes During the Evolution of Helicobacter pylori
Source: PLoS Genet. 2005 Oct 7;1(4):e43. doi: 10.1371/journal.pgen.0010043 (PMC1245399; doi:10.1371/journal.pgen.0010043)
Supplement: Supplementary file 5 [file pgen.0010043.sg005.pdf]

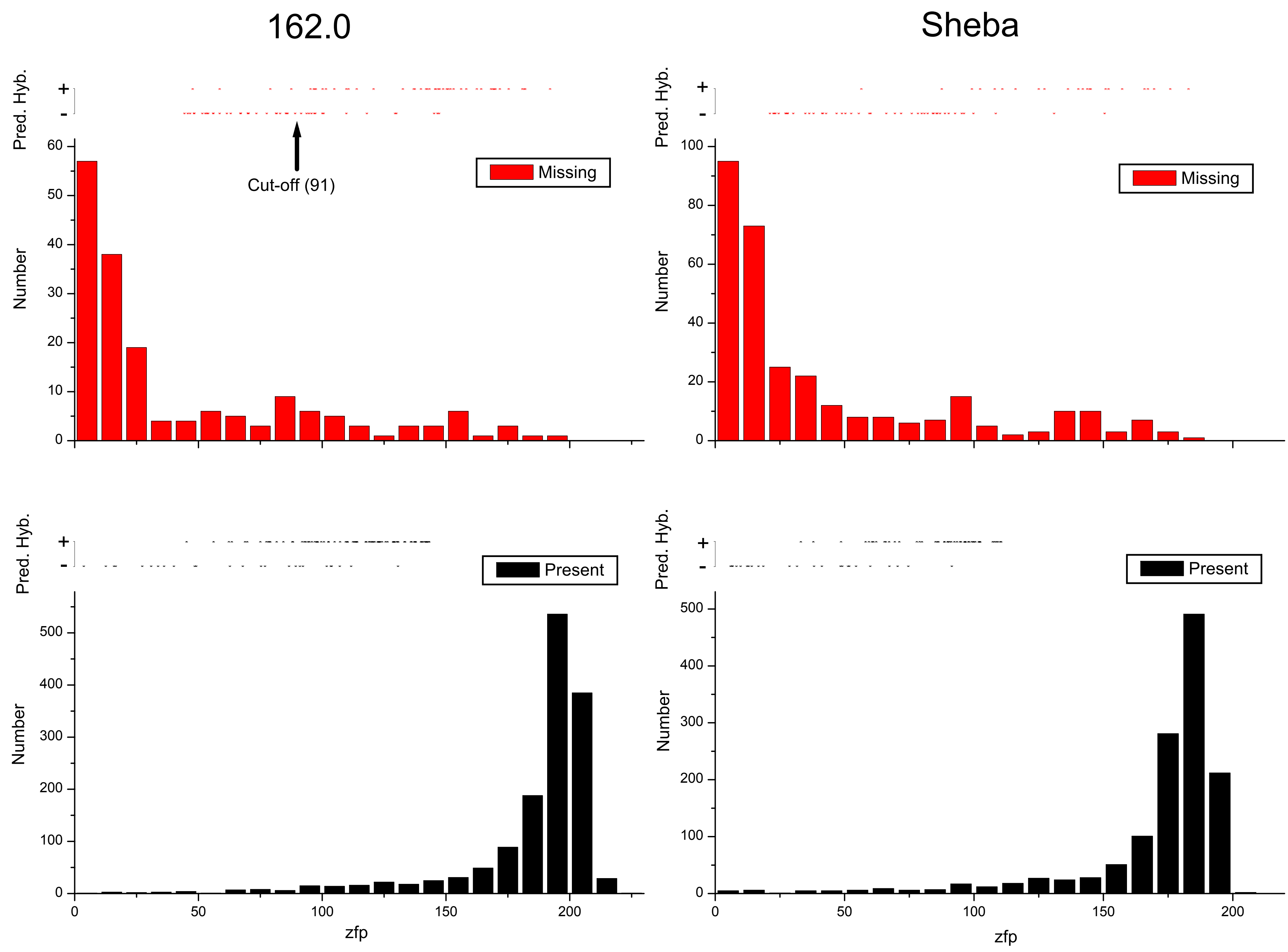
162.0
Sheba
Pred. Hyb.
+
-
Cut-off (91)
Missing
Present
Number
zfp
0
50
100
150
200
