# Supplementary material for: Gain and Loss of Multiple Genes During the Evolution of Helicobacter pylori
Source: PLoS Genet. 2005 Oct 7;1(4):e43. doi: 10.1371/journal.pgen.0010043 (PMC1245399; doi:10.1371/journal.pgen.0010043)
Supplement: Supplementary file 6 [file pgen.0010043.st006.pdf]

Table S6. Variable CDSs in *H. pylori* (sub)populations

Functional category	No. genes	Percentage that was variable or universally absent per population							
		hpAfrica2	hpAfrica1	hpEurope	hpAsia2	hspEAsia	hspMaori	hspAmerind	Amerind B
1. amino acid biosynthesis	45	4	0	0	0	0	0	0	2
2. purine, pyrimidine, nucleosides, nucleotides	45	2	0	0	0	0	0	7	0
3. fatty acid & phospholipid metabolism	29	7	10	7	7	3	0	3	3
4. cofactor, prosthetic group & carrier synthesis	72	1	1	0	0	3	0	4	11
5. central intermediary metabolism	16	0	0	0	0	0	0	0	0
6. energy metabolism	100	2	2	5	5	6	3	4	4
7. transport & binding proteins	107	4	2	5	1	2	5	4	13
8. DNA metabolism	131	34	33	45	34	34	25	46	36
9. transcription	18	0	0	0	0	0	0	0	0
10. protein synthesis	108	1	1	1	1	1	0	1	0
11. protein fate	65	0	0	0	0	0	0	0	0
12. regulatory functions	28	0	0	0	4	0	0	7	11
13. cell envelope	158	4	8	9	7	8	2	13	15
14. cellular processes / *cag* PAI	120	45	14	36	13	14	12	34	36
15. other categories / transposons	19	100	100	100	100	100	100	100	74
16/17. unknown function	610	30	32	33	31	31	26	33	29
Total:	1649	19% (322)	18% (294)	21% (353)	17% (286)	17% (291)	14% (236)	21% (359)	20% (334)

Note: Functional categories are according to Ref. [56]. The total does not correspond to the sum of all rows because 22 CDSs were assigned to two categories each. The raw numbers of CDSs are indicated in parentheses under Total.
