# Supplementary material for: Gain and Loss of Multiple Genes During the Evolution of Helicobacter pylori
Source: PLoS Genet. 2005 Oct 7;1(4):e43. doi: 10.1371/journal.pgen.0010043 (PMC1245399; doi:10.1371/journal.pgen.0010043)
Supplement: Supplementary file 7 [file pgen.0010043.st007.pdf]

Table S7. Accuracy, sensitivity, specificity and predictive value of hybridizations with the genomic sequences of Sheba and 162.0

Genome	Hybridization	zfp (No. of CDSs)			
		>91	<91	Predictive Value	Accuracy
Sheba	+	1263 (TP)	51 (FP)	Pos = 95.6%	93.3%
	-	58 (FN)	257 (TN)	Neg = 83.4%	
		Sensitivity = 95.6%	Specificity = 83.4%		
162.0	+	1417 (TP)	36 (FP)	Pos = 97.8%	95.8%
	-	32 (FN)	146 (TN)	Neg = 80.2%	
		Sensitivity = 97.8%	Specificity = 80.2%		

NOTE: TP, true positive; FP, false positive; TN, true negative; FN, false negative. Accuracy = (TN+TP)/(TN+TP+FN+FP).
Pos = Positive Predictive Value [TP/(TP+FP)]; Neg = Negative Predictive Value [TN/(FN+TN)].
Sensitivity [TP/(TP+FN)]; Specificity [TN/(FP+TN)]. Hybridization = observed hybridization with microarray.
